# IgE, α-Gal and atherosclerosis

**DOI:** 10.18632/aging.101894

**Published:** 2019-04-05

**Authors:** Jeffrey M. Wilson, Coleen A. McNamara, Thomas A.E. Platts-Mills

**Affiliations:** 1 Division of Allergy and Immunology, University of Virginia, Charlottesville, VA 22908, USA; 2 Division of Cardiology and Robert Berne Cardiovascular Center, University of Virginia, Charlottesville, VA 22908, USA

**Keywords:** atherosclerosis, Immunoglobulin E, galactose-alpha-1,3-galactose, red meat, risk factors

**Emerging evidence suggests a link between allergic disease and cardiovascular disease.** The idea that there could be a connection between ‘allergies’ and heart disease is not readily apparent. Nonetheless, over the past 20 years research in mice and humans has linked hematopoietic cells and soluble factors that mediate allergic disease with coronary artery disease (CAD) [1,2]. Specifically, mast cells [3] and elevated levels of the Immunoglobulin E (IgE) isotype [4] have been associated with the presence and/or severity of CAD. However, despite these associations, no specific allergens or IgE responses to specific allergens, have been implicated in CAD [4]. It was on this backdrop that a team of allergists and cardiologists collaborated to investigate the hypothesis that specific IgE to a novel oligosaccharide allergen present in mammalian meat could be a risk factor for CAD.

**Sensitization to α-Gal, an oligosaccharide allergen in mammalian meat, relates to tick bites.** Galactose-α-1,3-galactose (α-Gal) is a ‘blood-group-like’ antigen that is present as both glycoproteins and glycolipids in mammalian muscle and secretions (eg, milk) and is the causal epitope in a syndrome of IgE-mediated delayed anaphylaxis [5]. Because hypersensitivity reactions attributed to α-Gal may also include drugs (eg, the monoclonal antibody cetuximab), vaccines (eg, Varicella) or other blood products (eg, venom anti-sera), the name ‘α-Gal syndrome’ has been widely adopted [6]. There are several features of the α-Gal syndrome that distinguish it from traditional causes of anaphylaxis and food allergy. Notably, IgE sensitization to α-Gal relates to bites from certain hard ticks. In the United States there is solid evidence that *Amblyomma americanum* (the lone star tick) is a dominant ‘vector’ for inducing IgE to α-Gal, a finding that is supported by the strong overlap between cases of α-Gal syndrome and the established distribution of the lone star tick [5,7]. A critical point is that IgE sensitization to α-Gal has marked regional variability and is most common in areas of the southeast, lower Midwest and coastal Atlantic. Relatedly, it is also clear that the population prevalence of IgE sensitization to α-Gal is substantially greater than the prevalence of α-Gal syndrome that is recognized clinically. For example, in central Virginia it is estimated that ~20% of unselected children and adults have detectable titers of IgE to α-Gal, but the prevalence of clinically appreciated allergic symptoms attributed to α-Gal is thought to be at least 10-fold less [7]. Although high titers to α-Gal are associated with the risk of clinical symptoms, there are subjects who have high titers but nonetheless report eating mammalian meat without overt symptoms. This discrepancy raises important issues about co-factors that could affect the ‘penetrance’ of α-Gal syndrome in sensitized subjects. Equally, this discrepancy between the prevalence of sensitization versus *bona fide* α-Gal syndrome implies that there are likely many subjects who make IgE to α-Gal but nonetheless continue to consume mammalian products because they do not experience any allergic symptoms.

**Figure 1:**
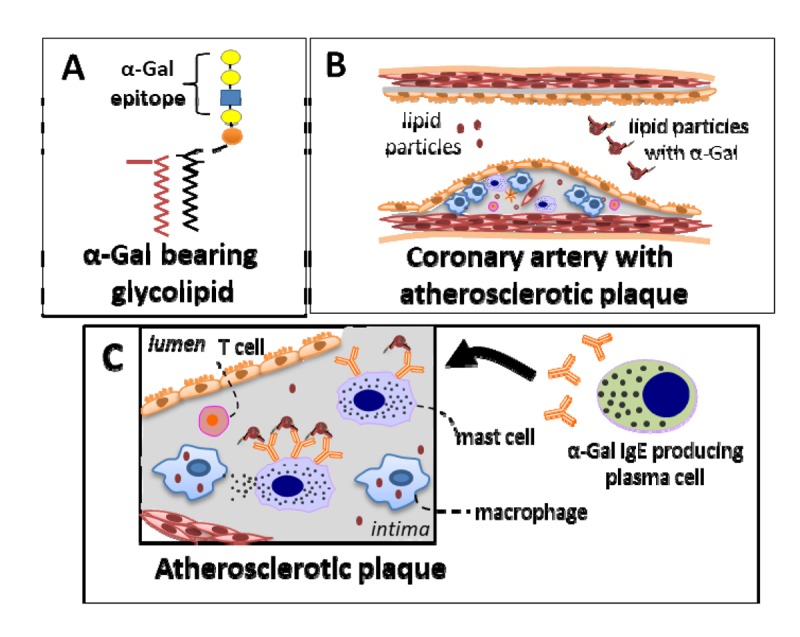
**Proposed model to explain the relationship between IgE sensitization to α-Gal and CAD.** The hypothesized relationship involves an interaction between a specific IgE immune response to α-Gal and dietary consumption of α-Gal-bearing glycolipids. (**A**) Representative depiction of a glycolipid with an α-Gal epitope. Such glycolipids are present in red blood cell membranes, muscle, fat and secretions of non-primate mammals. (**B)** Dietary lipids are absorbed through the intestinal epithelium and packaged into lipoprotein particles. Initially this involves chylomicrons before passing to smaller particles such as LDL or HDL that can subsequently deliver α-Gal epitopes to peripheral tissue where mast cells reside. Importantly, this can include mast cells within atherosclerotic plaques. (**C**) Specific IgE are bound to mast cells, including IgE specific to α-Gal, in subjects who are sensitized to the oligosaccharide, and thus α-Gal from dietary exposure could bind to and activate signal transduction via the high-affinity IgE receptor (Fc εR1). Relatively low levels of α-Gal exposure may be insufficient to induce mast cell degranulation, and thus not lead to overt allergic symptoms such as hives or anaphylaxis, but could nonetheless lead to chronic mast cell activation and pro-inflammatory effects.

**Evidence supporting a link between IgE to α-Gal and CAD.** The hypothesized connection between IgE to α-Gal and CAD was experimentally approached by a collaboration between cardiologists and allergists with a shared interest in understanding how immune regulation shapes health and disease. Adult subjects (n=118) from central Virginia who had been deemed to be at cardiovascular risk and had undergone heart catheterizations including advanced imaging with intravascular ultrasound (IVUS) formed the basis of the investigation [8]. An important caveat of the study is that these subjects were enrolled unrelated to any allergic history and dietary history was not collected at enrollment. When blood samples were assayed for IgE to α-Gal there were significantly greater amounts of atherosclerotic plaque in the 26% of the subjects that had detectable titers of IgE to α-Gal. Interestingly, this finding was most striking in the relatively younger subjects, ie 65 years or younger. In these younger subjects IVUS also revealed that the plaques had greater calcification, fibrofatty and necrotic content, findings consistent with higher risk plaques. The strength of the association between α-Gal IgE and CAD was stronger than the relationship between total IgE and CAD, or between IgE to unrelated inhalant or food allergens and CAD. The association also remained significant in multi-variate analysis that accounted for traditional CAD risk factors such as age, hypertension, diabetes and lipid levels. It is important to realize that both CAD and α-Gal-syndrome are common in individuals over 40 years old and many of the patients we see are over 60 (Figure 1).

**Building the case: α-Gal has a glycolipid form which could be the key to understanding delayed onset of allergic reactions and a link with CAD.** The recent report, which suggests the possibility that IgE to α-Gal represents a tick-related risk factor for CAD, is provocative but nonetheless only represents an association at the current time. However, there are additional elements that support the possibility of a mechanistic link. One is the realization that the prevalence of CAD, like α-Gal sensitization, also has marked regional variation and in the United States is most common in the southeast. In fact, there is significant overlap in the areas of the country where CAD and α-Gal syndrome are most common. Additionally, unique among major allergens, α-Gal can be present in a glycolipid form. There are many reasons to think that the glycolipid form of the allergen is the key to explain the characteristic delay of 3-6 hours that occurs with ingestion of mammalian meat, kinetics which are much slower than the onset of symptoms that result from parenteral exposure to α-Gal-containing allergen (ie, cetuximab or varicella). Of course this ‘glycolipid hypothesis’ suggests another important tie with CAD given that it is well established that serum levels of low-density-lipoprotein (LDL) cholesterol are a risk factor for CAD and LDL deposits are found within lesions of atherosclerotic plaques. A working model to explain the connection between α-Gal and CAD posits that subjects who consume mammalian products containing α-Gal, and also make IgE to α-Gal, have chronic inflammation in the walls of coronary arteries that relates to this interaction between an antigen in food and a ‘unique’ immune response that recognizes that antigen. Further epidemiological and mechanistic studies will be imperative to further understand the relationship between IgE to α-Gal and CAD.
